# Putrescine Depletion in *Leishmania donovani* Parasites Causes Immediate Proliferation Arrest Followed by an Apoptosis-like Cell Death

**DOI:** 10.3390/pathogens14020137

**Published:** 2025-02-02

**Authors:** Julia Johnston, Jonathan Taylor, Surbhi Nahata, Angelica Gatica-Gomez, Yvette L. Anderson, Sophia Kiger, Thong Pham, Kayhan Karimi, Jasmin-Faith Lacar, Nicola S. Carter, Sigrid C. Roberts

**Affiliations:** School of Pharmacy, Pacific University, Hillsboro, OR 97123, USA; juliajohnston@pacificu.edu (J.J.); taylorjo@pacificu.edu (J.T.); snahata@uvm.edu (S.N.); gati3896@pacificu.edu (A.G.-G.); yvette.l.anderson@gsk.com (Y.L.A.); sophiakiger@pacificu.edu (S.K.); pham3316@pacificu.edu (T.P.); kari9098@pacificu.edu (K.K.); laca4839@pacificu.edu (J.-F.L.); cartern@pacificu.edu (N.S.C.)

**Keywords:** *Leishmania*, polyamines, apoptosis, replication, mitochondria, starvation, ornithine decarboxylase, spermidine synthase

## Abstract

The polyamine pathway in *Leishmania* parasites has emerged as a promising target for therapeutic intervention, yet the functions of polyamines in parasites remain largely unexplored. Ornithine decarboxylase (ODC) and spermidine synthase (SPDSYN) catalyze the sequential conversion of ornithine to putrescine and spermidine. We previously found that *Leishmania donovani* Δ*odc* and Δ*spdsyn* mutants exhibit markedly reduced growth in vitro and diminished infectivity in mice, with the effect being most pronounced in putrescine-depleted Δ*odc* mutants. Here, we report that, in polyamine-free media, ∆*odc* mutants arrested proliferation and replication, while ∆*spdsyn* mutants showed a slow growth and replication phenotype. Starved ∆*odc* parasites also exhibited a marked reduction in metabolism, which was not observed in the starved ∆*spdsyn* cells. In contrast, both mutants displayed mitochondrial membrane hyperpolarization. Hallmarks of apoptosis, specifically DNA fragmentation and membrane modifications, were observed in Δ*odc* mutants incubated in polyamine-free media. These results show that putrescine depletion had an immediate detrimental effect on cell growth, replication, and mitochondrial metabolism and caused an apoptosis-like death phenotype. Our findings establish ODC as the most promising therapeutic target within the polyamine biosynthetic pathway for treating leishmaniasis.

## 1. Introduction

Leishmaniasis, a neglected tropical disease, with over one billion people at risk for infection living in endemic areas in nearly 90 countries across Africa, South-East Asia, the Middle East, Europe, and Central and South America. Annually, approximately 1 million new cases and 70,000 deaths are reported [1,2,3,4]. The risk of infection significantly increases in impoverished communities, and recent outbreaks have been fueled by factors such as human migration, civil unrest, and war [1,5,6,7,8]. Additionally, environmental issues like deforestation, urbanization associated with poor sanitary conditions, overcrowding or lack of infrastructure, and climate change have contributed to the rising incidence of cases. Moreover, leishmaniasis is now considered to be endemic in the United States [1,3,8,9,10,11,12,13].

*Leishmania* parasites have a dimorphic life cycle, existing as flagellated promastigotes in sand flies and non-flagellated amastigotes in mammals, primarily residing in macrophages [2,4,10]. Leishmaniasis in humans, caused by over 20 different species of *Leishmania*, manifests mainly as cutaneous and visceral forms. Cutaneous leishmaniasis (CL) leads to ulcerative skin lesions, with estimates ranging from 600,000 to 1 million new cases each year [3]. In contrast, visceral leishmaniasis (VL), caused by *L. donovani* and *L. infantum*, is predominantly fatal if untreated and is the second leading cause of mortality among parasitic diseases. An estimated 50,000–90,000 new infections occur annually, although underreporting remains significant [3]. VL affects internal organs and presents symptoms such as fever and weight loss [2,3,7,10].

Currently, no vaccines exist to prevent leishmaniasis in humans, and treatment options are limited, often with severe side effects and growing drug resistance complicating care [2,10,14,15,16,17,18,19]. Furthermore, the persistence of *Leishmania* parasites post-treatment has spurred ongoing research into the mechanisms behind their resilience [20,21,22,23]. Together, the lack of ideal treatment options, the absence of a vaccine, and the increasing incidence and spread of the disease underscore the urgent need to identify new therapeutic targets.

Notably, polyamine biosynthesis has already been clinically validated as a treatment target in the related pathogen *Trypanosoma brucei gambiense* [24,25,26,27]. These ubiquitous and essential cations play a critical role in various cellular processes, including growth, differentiation, and macromolecular synthesis [28,29,30,31,32]. A key inhibitor in this pathway, D,L-α-difluoromethylornithine (DFMO, eflornithine) effectively targets ornithine decarboxylase (ODC), the enzyme responsible for synthesizing the polyamine putrescine. DFMO has demonstrated remarkable success in treating African sleeping sickness caused by *Trypanosoma brucei gambiense* [24,25,27]. DFMO is also active against *Leishmania* in vitro and in murine and hamster infectivity models, and recent studies have highlighted the importance of the polyamine biosynthetic pathway as a potential therapeutic target in *Leishmania* [27,28,33,34,35,36].

**Figure 1:**
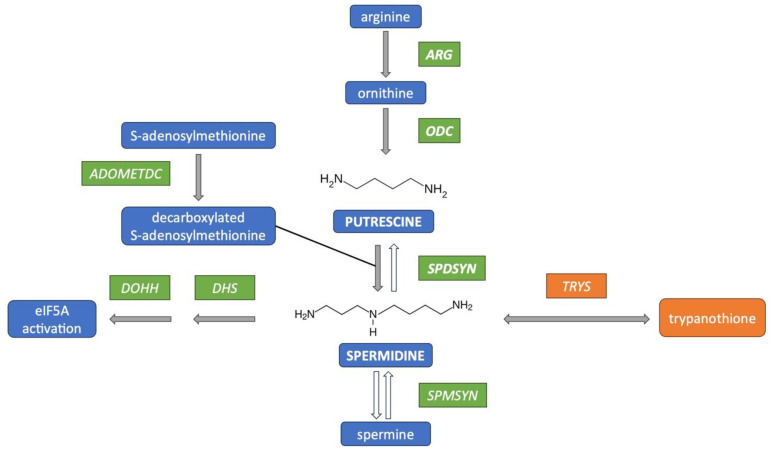
Polyamine biosynthetic pathway in *Leishmania* parasites. The polyamine biosynthetic pathway in *Leishmania* is depicted with gray arrows. This pathway illustrates the sequential conversion of arginine to ornithine, putrescine, and spermidine, catalyzed by arginase (ARG), ornithine decarboxylase (ODC), and spermidine synthase (SPDSYN), respectively. S-adenosylmethionine decarboxylase (ADOMETDC) generates decarboxylated S-adenosylmethionine, which serves as the aminopropyl donor for spermidine synthesis. The two polyamines produced in *Leishmania*, putrescine and spermidine, are shown in uppercase. Unique to trypanosomatids is the reversible formation of trypanothione, catalyzed by the bidirectional enzyme trypanothione synthetase/amidase (TRYS) in *Leishmania*. The modification and activation of eukaryotic translation initiation factor 5A (eIF5A) by deoxyhypusine synthase (DHS) and deoxyhypusine hydroxylase (DOHH) occur in both *Leishmania* parasites and the human host. White arrows indicate the spermine synthase (SPMSYN) reaction and the simplified back-conversion pathway that is present in the mammalian host but absent in *Leishmania*.

The polyamine biosynthetic pathway in *Leishmania* consists of four enzymes: arginase (ARG), ornithine decarboxylase (ODC), spermidine synthase (SPDSYN), and S-adenosylmethionine decarboxylase (ADOMETDC) (Figure 1). ARG converts the essential amino acid arginine to ornithine, which is directly channeled into polyamine biosynthesis. ODC then converts ornithine to putrescine, and SPDSYN produces spermidine, a vital metabolite involved in the hypusination and activation of eukaryotic translation initiation factor 5A (eIF5A) in both the parasite and host [37,38,39]. Unique to trypanosomatids, spermidine conjugates with glutathione to form trypanothione, which is essential for redox balance and oxidative stress defense [40,41]. Trypanothione synthetase/amidase (TRYS) catalyzes its synthesis and hydrolysis [42]. Unlike humans, *Leishmania* neither produces spermine nor has a polyamine back-conversion pathway [43].

We previously generated gene deletion mutants for ODC (∆*odc*) and SPDSYN (∆*spdsyn*) in *L. donovani* using targeted gene replacement strategies [43,44,45,46]. Characterization of these mutants revealed that both enzymes are essential for polyamine biosynthesis, as the conditionally lethal null mutants depend on supplementation with putrescine or spermidine for growth. The ∆*odc* mutants exhibit profoundly reduced infectivity compared to wild-type parasites, while the ∆*spdsyn* mutants show a less pronounced yet substantial decrease in infectivity [44,45]. The inability of ∆*odc* mutants to establish infections suggests that putrescine is unavailable to intracellular parasites, a hypothesis supported by the rapid conversion of arginine to spermine in macrophages [47] and the typically low levels of putrescine in differentiated mammalian cells [48,49]. Our findings, combined with evidence that DFMO reduces infectivity in mice and hamsters [50,51,52], validate ODC as a potential therapeutic target in *Leishmania*. Notably, the structure of the leishmanial ODC features a unique N-terminal extension not found in the human enzyme [53], and both computer modeling and inhibitor studies demonstrate that the enzyme is a druggable target [53,54,55,56,57,58,59].

Although most research has focused on the promastigote stage, polyamines are clearly essential for the amastigote stage of *Leishmania*. Both ODC and SPDSYN are expressed in amastigotes [44,60], and gene deletion mutants exhibit significantly reduced infectivity [44,45]. Additionally, inhibitors targeting polyamine pathway enzymes are effective against intracellular amastigotes both in vitro and in rodent infectivity models [28].

Alterations in polyamine metabolism have been observed in *Leishmania* strains that are resistant to standard anti-leishmanial drugs, likely through their impact on trypanothione biosynthesis, a key component of the parasite’s antioxidant defense [61,62,63]. Gene amplification or increased ODC expression has been observed in antimony-resistant strains [61,64,65,66], while elevated arginine, ornithine, and spermidine levels are associated with miltefosine resistance [67,68]. In contrast, reduced putrescine levels occur in pentamidine-resistant strains [69,70]. Combining anti-leishmanial drugs with polyamine pathway inhibitors has the potential to restore drug sensitivity or prevent resistance development [28].

Notable differences between the growth phenotypes of the ∆*odc* and ∆*spdsyn* mutants were also observed in vitro [71]. In the ∆*odc* mutants, putrescine depletion leads to cell rounding, immediate cessation of proliferation, and loss of viability, whereas putrescine-rich ∆*spdsyn* mutants display an intermediate proliferation phenotype and can persist in a quiescent-like state from five to six weeks before cell death occurs. Contrary to the long-standing belief that putrescine’s sole function is as precursor for spermidine synthesis [43] (Jiang et al., 1999), these findings suggest that it is also crucial for parasite growth and infectivity. However, the functions of putrescine remain largely unexplored, highlighting the need for further investigation into its role in cellular processes. The ∆*odc* and ∆*spdsyn* mutants serve as ideal tools due to their distinct intracellular polyamine dynamics. Specifically, putrescine levels deplete rapidly in ∆*odc* mutants incubated in polyamine-free media, while they accumulate in ∆*spdsyn* mutants under the same conditions [71]. In contrast, spermidine levels remain low but stable in both cell lines [71].

In this study, we investigated the effects of polyamine withdrawal on cell growth, metabolism, and death in *L. donovani* ∆*odc* and ∆*spdsyn* mutant cell lines. Our findings demonstrate that ∆*odc* mutants exhibited rapid arrest in proliferation and replication, alongside significant metabolism impairment, while ∆*spdsyn* mutants displayed a much more moderate phenotype. Both mutants showed hyperpolarization of the mitochondrial membrane, but only the ∆*odc* mutants displayed hallmarks of apoptosis, specifically DNA fragmentation and membrane modifications. These results underscore the critical role of putrescine in cellular function and highlight ODC as a promising therapeutic target in the polyamine biosynthetic pathway for the treatment of leishmaniasis.

## 2. Materials and Methods

### 2.1. Materials

Dulbecco’s Modified Eagle Medium and chicken serum were procured from Thermo Fisher Scientific (Waltham, MA, USA). Antibiotics, including hygromycin, neomycin, and puromycin, were obtained from InvivoGen (San Diego, CA, USA). Resazurin was purchased from VWR International (Radnor, PA, USA), putrescine, spermidine, and carbonyl cyanide m-chlorophenyl hydrazone (CCCP) were sourced from MilliporeSigma (Burlington, MA, USA), and 5,5′,6,6′-tetrachloro-1,1′,3,3′-tetraethyl benzimidazolyl carbocyanine iodide (JC-1) was bought from Cayman Chemical (Ann Arbor, MI, USA). The BrdU Cell Proliferation ELISA Kit (colorimetric) was acquired from Abcam (Cambridge, UK) and the In Situ Cell Death Detection Kit, Fluorescein, was purchased from Roche (Basel, Switzerland). Propidium iodide (PI) was obtained from Cell Signaling Technology (Danvers, MA, USA), and FITC Annexin V was purchased from BioLegend (San Diego, CA, USA).

### 2.2. Cell Lines and Culture Conditions

Promastigote parasites were cultured at 27 °C in a completely defined Dulbecco’s Modified Eagle Medium optimized for *Leishmania* promastigotes. In this medium, fetal bovine serum was substituted with chicken serum to prevent polyamine oxidase-mediated toxicity (DME-L CS) [46,71,72]. All genetically modified parasites originated from the wild-type (WT) LdBob strain of *L. donovani* [73], which was initially provided by Dr. Stephen M. Beverley (Washington University, St. Louis, MO). The Δ*odc* and Δ*spdsyn* mutants were previously created using targeted gene replacement methods [44,45] and contain the hygromycin phosphotransferase and neomycin phosphotransferase drug resistance genes (Δ*odc*) or the hygromycin phosphotransferase and puromycin acetyltransferase drug resistance genes (Δ*spdsyn*). The Δ*odc* cell line was routinely grown in the presence of 100 μM putrescine, 50 μg/mL hygromycin, 20 μg/mL neomycin, and the Δ*spdsyn* cell line was cultured in 100 μM spermidine, 50 μg/mL hygromycin, 10 μg/mL puromycin, unless otherwise specified.

### 2.3. Proliferation Assay

All three cell lines were washed three times in phosphate-buffered saline (PBS) to remove any residual polyamines. Centrifugation steps were carried out at 1452 relative centrifugal force (RCF) for 10 min at room temperature. For the initial proliferation curve comparing polyamine starvation among cell lines, wild-type cells were incubated in polyamine-free media lacking both drugs and polyamine supplementation. The ∆*odc* mutants were incubated in either polyamine-free media or media supplemented with 100 µM putrescine, while ∆*spdsyn* mutants were grown in polyamine-free media or media supplemented with 100 µM spermidine. For the proliferation curve examining polyamine supplementation in wild-type cells, parasites were incubated in polyamine-free media or in media supplemented with 500 µM putrescine, or 500 µM spermidine, or a combination of 500 µM putrescine and 500 µM spermidine. All cell lines were seeded at a density of 5 × 10^5^ cells/mL on day 0, and their growth was monitored over a period of 11 days. Cell counting was performed using a MacsQuant 10 flow cytometer (Miltenyi Biotec, Bergisch Gladbach, Germany).

### 2.4. Replication Assay

Bromodeoxyuridine incorporation was measured using a BrdU Cell Proliferation ELISA Kit (colorimetric) from Abcam. This assay was used to assess DNA replication in wild-type parasites and in mutant cell lines that were supplemented with polyamines or incubated in polyamine-free media.

Parasites were washed three times in PBS and resuspended at a density of 5 × 10^6^ cells/mL in the following media conditions: wild-type, ∆*odc*, and ∆*spdsyn* parasites in polyamine-free media; ∆*odc* mutants in media supplemented with 100 µM putrescine; and ∆*spdsyn* mutants in media supplemented with 100 µM spermidine. After overnight incubation at 27 °C, 1 × 10^8^ parasites were harvested, centrifuged, and seeded in triplicate at 1 × 10^6^ cells/100 µL in 96-well plates with fresh media corresponding to their initial conditions. Centrifugation steps were carried out at 1452 RCF for 10 min at room temperature.

Cells were then treated according to the manufacturer’s protocol. Briefly, 20 µL of 1× BrdU was added to each well, except the wild-type negative control wells, followed by overnight incubation at 27 °C. Cells were fixed and incubated with an anti-BrdU monoclonal detector antibody, followed by Peroxidase Goat Anti-Mouse IgG Conjugate and TMB Peroxidase Substrate incubation. After adding the Stop Solution, absorbance was measured at 450 nm using a BioTek Synergy H1 Multimode Reader (Agilent, Santa Clara, CA, USA).

### 2.5. Metabolism Assay

Metabolic activity was determined by measuring the conversion of resazurin into the fluorescent compound resorufin.

Parasites were washed three times in PBS before incubation in the appropriate media. Centrifugation steps were carried out at 1452 RCF for 10 min at room temperature. Wild-type cells were incubated in polyamine-free media. The ∆*odc* mutants were cultured either in polyamine-free media or media supplemented with 100 µM putrescine, while the ∆*spdsyn* mutants were incubated in either polyamine-free media or media supplemented with 100 µM spermidine. Wild-type parasites and mutant cells in supplemented media were seeded at a concentration of 5 × 10^5^ cells/mL on day 0. To ensure sufficient cell material for the assay, ∆*odc* mutants in polyamine-free media were seeded at a higher concentration of 1 × 10^7^ cells/mL, and ∆*spdsyn* mutants in polyamine-free media were seeded at 2 × 10^6^ cells/mL.

Resazurin fluorescence and cell numbers were measured on days 3 and 4 of starvation, during the log phase of cell growth, when metabolic activity is expected to be optimal. Cells (1 × 10^7^) were harvested, centrifuged at 1452 RCF for 10 min at room temperature, and resuspended in 1 mL of the same media. Cells were then counted in triplicate in a 96-well plate with 100 µL samples per well using the MACSQuant flow cytometer. Following this, 10 µL of 44 µM resazurin was added to each well, and plates were incubated at 27 °C for four hours. Resorufin fluorescence was measured at 554_Ex_–593_Em_ nm using the BioTek Synergy H1 Multimode Reader. Metabolic activity per cell was calculated by dividing the resorufin fluorescence by the number of cells in each sample.

### 2.6. Assessment of Mitochondrial Membrane Potential

The mitochondrial membrane potential (ΔΨm) was assessed using the cationic dye JC-1. This dye aggregates within mitochondria with an intact mitochondrial membrane potential, emitting red fluorescence at 590 nm, whereas in cells with depolarized mitochondrial membranes, JC-1 remains cytosolic and monomeric, displaying green fluorescence at 530 nm.

Parasites were washed three times in PBS before incubation in the appropriate media. Centrifugation steps were carried out at 1452 RCF for 10 min at room temperature. Wild-type cells were incubated in polyamine-free media. The ∆*odc* mutants were cultured either in polyamine-free media or media supplemented with 100 µM putrescine, while the ∆*spdsyn* mutants were incubated in either polyamine-free media or media supplemented with 100 µM spermidine. Wild-type parasites and mutant cells in supplemented media were seeded at a concentration of 3 × 10^5^ cells/mL on day 0. To ensure sufficient cell material for the assay, ∆*odc* mutants in polyamine-free media were seeded at a higher concentration of 5 × 10^6^ cells/mL, and ∆*spdsyn* mutants in polyamine-free media were seeded at 1 × 10^6^ cells/mL.

Samples were collected on days 3, 4, and 8, washed once with PBS supplemented with 158 µg/mL glucose (PSG), and resuspended in 1 mL PSG. Centrifugation steps were carried out at 6021.1 RCF for 5 min at room temperature. As a control, wild-type cells were treated with the mitochondrial uncoupler CCCP at 75 µM for 10 min at 27 °C. All samples were stained with 0.22 mM JC-1 (except an unstained wild-type control), incubated for one hour at 27 °C, centrifuged, and resuspended in 500 µL PSG. Analysis was performed on the MACSQuant flow cytometer using an excitation laser at 488 nm, 30 mW. Emission signals were collected using the FITC B1 (525/50 nm) and PE B2 (585/40 nm) detectors to capture green and red fluorescence, respectively. Flow cytometer data were analyzed using FlowJo^TM^ v10 (BD Life Sciences, Franklin Lakes, NJ, USA), and the 590:530 fluorescence ratio was calculated for each data point.

### 2.7. DNA Fragmentation Assay

DNA fragmentation was assessed using the In Situ Cell Death Detection Kit (Roche), which is based on the Terminal deoxynucleotidyl transferase (TdT) dUTP Nick End Labeling (TUNEL) method and detects free 3′-hydroxyl ends generated by endonuclease degradation.

Parasites were washed three times in PBS before incubation in the appropriate media. Centrifugation steps were carried out at 1452 RCF for 10 min at room temperature. Wild-type cells were incubated in polyamine-free media. The ∆*odc* mutants were cultured either in polyamine-free media or media supplemented with 100 µM putrescine, while the ∆*spdsyn* mutants were incubated in either polyamine-free media or media supplemented with 100 µM spermidine. Wild-type parasites and mutant cells in supplemented media were seeded at a concentration of 5 × 10^5^ cells/mL on day 0. To ensure sufficient cell material for the assay, ∆*odc* mutants in polyamine-free media were seeded at a higher concentration of 5 × 10^6^ cells/mL, and ∆*spdsyn* mutants in polyamine-free media were seeded at 1 × 10^6^ cells/mL.

Samples were collected on days 4, 7, and 10 and processed according to the manufacturer’s instructions. Briefly, 1 mL cells were harvested and washed in PBS before being fixed in a 2% paraformaldehyde (PFA) solution in PBS for 60 min. Centrifugation steps were carried out at 6021.1 RCF for 5 min at room temperature. Following PFA removal, cells were permeabilized with the kit’s permeabilization buffer, washed with PBS, and incubated with the TUNEL reaction mixture for 1 h at 37 °C. After incubation, the cells were washed again and analyzed using the MACSQuant flow cytometer. Single-channel trace files generated from the flow cytometer were overlaid and analyzed in FlowJo^TM^ v10 using the “Compare population” function to determine Overton % positive cell populations. Stained populations were compared with a mixed stock of unlabeled control cells.

### 2.8. Membrane Modifications Assay

Membrane modifications were evaluated using FITC-Annexin V (Biolegend) and PI (Cell Signaling Technology) staining. Annexin V binds to phosphatidylserine exposed on the outer leaflet of the plasma membrane during early apoptosis, while PI penetrates cells with compromised membranes, marking late apoptotic or necrotic cells.

Parasites were washed three times in PBS before incubation in the appropriate media. Centrifugation steps were carried out at 1452 RCF for 10 min at room temperature. Wild-type cells were incubated in polyamine-free media. The ∆*odc* mutants were cultured either in polyamine-free media or media supplemented with 100 µM putrescine, while the ∆*spdsyn* mutants were incubated in either polyamine-free media or media supplemented with 100 µM spermidine. Samples were collected on days 3 or 4, 7, and 14. Approximately 600 µL of cell culture was harvested and washed twice with 1 mL of PBS before being resuspended in 200 µL of Annexin V binding buffer (10 mM HEPES, 150 mM NaCl, 2.5 mM CaCl_2_ in H_2_O) containing 5 µL of 90 ug/mL Annexin V and 5 µL of 10 mg/mL PI. Centrifugation steps were carried out at 6021.1 RCF for 5 min at room temperature. The cells were then incubated at room temperature for 30 min, during which they were protected from light and then analyzed using the MACSQuant flow cytometer. Appropriate single-stained and unstained controls were prepared and treated identically.

Scatterplots generated using flow cytometry data were analyzed using FlowJo^TM^ v10 with quadrant gating applied according to the manufacturer’s guidelines. Percentages of cells within each quadrant were exported to GraphPad Prism v10 (GraphPad Prism, Boston, MA, USA) for data analysis.

### 2.9. Data Visualization and Statistical Analysis

Data visualization and statistical analysis was conducted using GraphPad Prism v10. Error bars in the graphs represent standard deviations. Statistical analysis was conducted using ANOVA, and statistical comparisons between group means were considered significant at *p* < 0.05.

## 3. Results

### 3.1. Extended Polyamine Starvation Exposes Distinct Growth Patterns in Parasites

A prior study demonstrated that ∆*odc* parasites do not proliferate in polyamine-free media, while ∆*spdsyn* parasites exhibit only slow growth under these conditions over a 7-day period [71]. In the current study, we extended these observations to 11 days of polyamine-free incubation to monitor the growth phenotype further and assess whether ∆*spdsyn* mutants eventually achieve cell numbers comparable to wild-type or polyamine-supplemented parasites.

**Figure 2:**
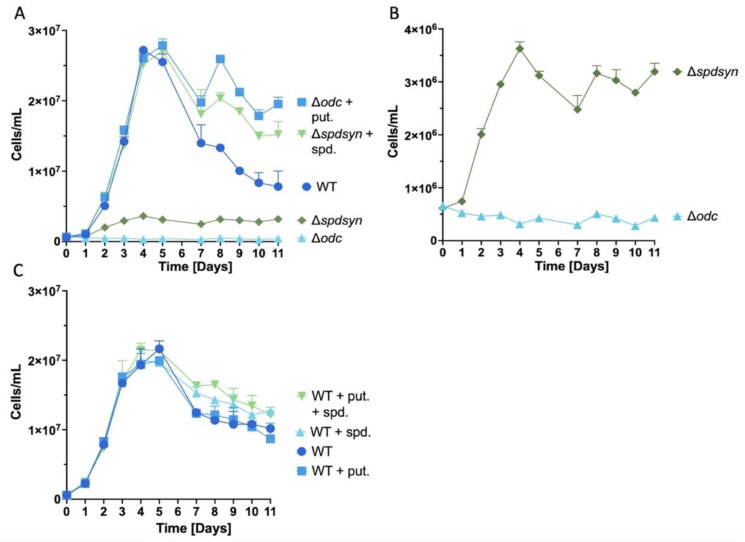
Proliferation of wild-type and mutant parasites in response to polyamine availability. Parasite proliferation was monitored over 11 days using a flow cytometer. (**A**) Proliferation of wild-type (WT) cells in polyamine-free media (dark blue circles), ∆*odc* mutants in media with 100 µM putrescine (blue squares), ∆*spdsyn* mutants in media with 100 µM spermidine (green triangles), as well as ∆*odc* (light blue triangles) and ∆*spdsyn* mutants (dark green diamonds) in polyamine-free media. (**B**) Growth of the ∆*odc* (light blue triangles) and ∆*spdsyn* (dark green diamonds) mutants grown in polyamine-free media is shown to allow for a better comparison of the cellular proliferation rate between the two mutants. (**C**) Growth of wild-type parasites in polyamine-free media (dark blue circles), in media supplemented with 500 µM putrescine (blue squares), 500 µM spermidine (light blue triangles), or a combination of 500 µM putrescine and 500 µM spermidine (light green triangles). Three experiments were conducted in technical triplicate (*n* = 3) for each experimental design, as illustrated in panels (**A**–**C**). Consistent results were observed across all experiments, and one representative experiment from each design is shown. The two other experiments for each design are shown in Figure S1.

During the first 7 days, all cell lines displayed growth trends consistent with previously published observations [71] (Figure 2A). The ∆*spdsyn* mutants incubated in polyamine-free media showed a maximum density of ~4 × 10^6^ cells/mL—notably higher than the ∆*odc* mutants, but almost ten-fold lower than wild-type or supplemented cultures. After reaching this plateau, ∆*spdsyn* mutants sustained this low maximum cell number without further growth between days 4–11 (Figure 2B). In contrast, ∆*odc* mutants in polyamine-free media showed no proliferation, maintaining a stable cell count around 3–5 × 10^5^ cells/mL throughout the 11-day experiment (Figure 2B).

Both wild-type cells and mutants cultured in polyamine-supplemented media exhibited similar growth patterns through logarithmic and stationary phases (Figure 2A). By day 4 or 5, they peaked at approximately 3 × 10^7^ cells/mL, followed by a gradual decline likely due to general nutrient depletion and overgrowth. However, in week two, notable differences emerged between the wild-type parasites and supplemented mutants (Figure 2A). Wild-type cell counts decreased continuously from a peak of approximately 2.5 × 10^7^ cells/mL to below 1 × 10^7^ cells/mL by day 11 (~60% loss in cell density). In contrast, cell counts for the supplemented mutants dipped slightly, but stabilized around 2 × 10^7^ cells/mL between days 7 to 11 (~20% loss in cell density).

Because previous research demonstrated higher intracellular polyamine levels in supplemented ∆*odc* and ∆*spdsyn* mutants compared to wild-type parasites [71], we aimed to investigate whether polyamine supplementation could stabilize cell concentration and prolong survival, as observed in the supplemented mutants during week 2 (Figure 2A). To examine this, wild-type parasites were incubated in media enriched with 500 µM putrescine, 500 µM spermidine, or a combination of both (500 µM putrescine plus 500 µM spermidine). The growth patterns of wild-type parasites were similar regardless of the supplement conditions (Figure 2C) and did not show the distinct differences observed between wild-type parasites and supplemented mutants in Figure 2A.

To summarize, we show that ∆*spdsyn* mutants achieved higher cell densities than ∆*odc* mutants, but plateaued at levels far below wild-type or supplemented cultures. Supplemented mutant cells maintained stable counts through week two, while wild-type cells declined, showing a distinct difference in growth dynamics.

### 3.2. Putrescine Is Essential for DNA Synthesis and Replication

**Figure 3:**
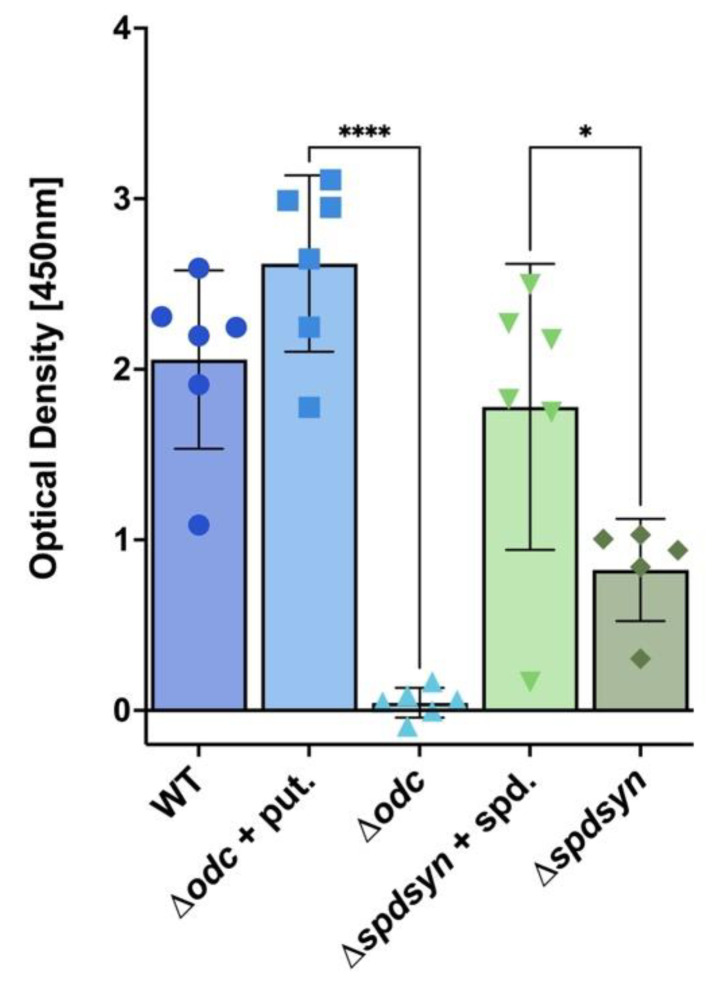
DNA synthesis in wild-type and mutant cell lines. DNA synthesis was assessed by measuring BrdU incorporation. The optical density, representative of BrdU incorporation levels, is displayed for wild-type cells (WT), ∆*odc* mutants supplemented with 100 µM putrescine, ∆*spdsyn* mutants supplemented with 100 µM spermidine, and ∆*odc* and ∆*spdsyn* mutants incubated in polyamine-free media. The experiment was performed twice in biological triplicate (*n* = 6). Statistical significance is represented as follows: * *p* ≤ 0.05, and **** *p* ≤ 0.0001.

To investigate whether polyamine depletion affects DNA replication and potentially contributes to the growth arrest of ∆*odc* parasites, we measured BrdU incorporation as a marker of DNA synthesis. Wild-type and supplemented mutant cells exhibited proficient BrdU incorporation, with no significant differences between them (Figure 3). In contrast, ∆*odc* mutants grown in polyamine-free media showed virtually no BrdU incorporation, which was comparable to the no-cell control and significantly less than that of ∆*odc* mutants in 100 µM putrescine (*p* < 0.0001) (Figure 3). Although ∆*spdsyn* mutants in polyamine-free media displayed some BrdU incorporation, it was significantly less than that of mutants in 100 µM spermidine (*p* = 0.0242) (Figure 3). The lack of DNA synthesis in ∆*odc* mutants and the limited DNA synthesis in ∆*spdsyn* mutants grown in polyamine-free media corresponded to their respective growth phenotypes, as illustrated in Figure 2A,B.

### 3.3. Putrescine Depletion Reduces Metabolism

**Figure 4:**
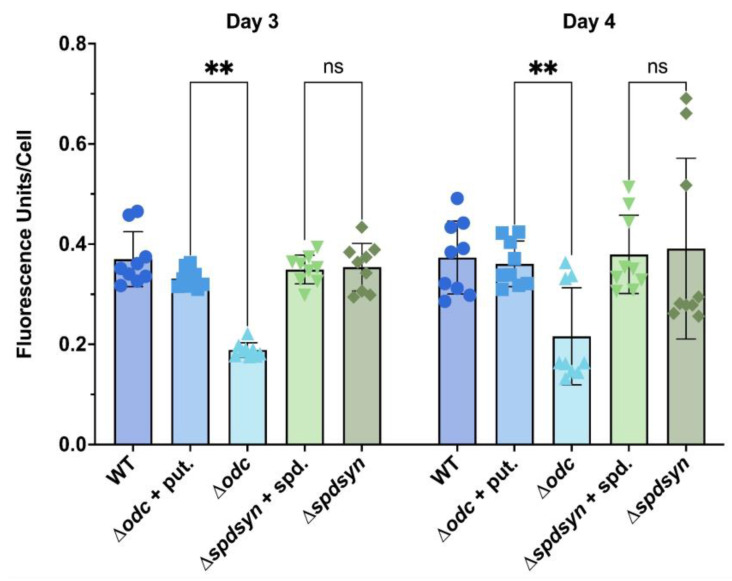
Metabolic activity of wild-type and mutant cell lines. Fluorescence units per cell, reflecting the conversion of resazurin to resorufin as a measure of metabolic activity, are shown. Fluorescence was measured in wild-type parasites (WT), ∆*odc* mutants incubated in either 100 µM putrescine-supplemented or polyamine-free media, and ∆*spdsyn* mutants incubated in either 100 µM spermidine-supplemented or polyamine-free media. The experiment was performed three times in technical triplicate (*n* = 9). Statistical significance is represented as follows: ns (not significant) and ** *p* ≤ 0.01.

To investigate if polyamine deprivation reduces metabolic activity, the conversion of resazurin to the fluorescent compound resorufin via intracellular reductases was measured. Measurements were taken on days 3 and 4, when parasites underwent robust logarithmic growth (Figure 2A). No statistically significant differences were observed between wild-type cells and supplemented mutants (Figure 4). However, the results show a significant reduction in metabolism in the ∆*odc* mutants incubated in polyamine-free media compared to those incubated in 100 µM putrescine (*p* = 0.0023 for day 3, *p* = 0.0019 for day 4) as well as to wild-type and ∆*spdsyn* parasites (Figure 4). In contrast, no significant difference in resazurin conversion was observed in ∆*spdsyn* mutants incubated in polyamine-free media compared to those grown in 100 µM spermidine (Figure 4). In summary, resazurin conversion showed that wild-type cells, supplemented ∆*odc* mutants, and ∆*spdsyn* mutants (in both polyamine-free and -supplemented media) exhibited similar metabolic activity on days 3 and 4, while the metabolism was significantly reduced in ∆*odc* mutants under polyamine deprivation.

### 3.4. Polyamine Depletion Affects Mitochondrial Membrane Potential

To assess whether the mitochondrial potential is compromised in polyamine-starved mutant cell lines, we employed JC-1, a commonly used dye for evaluating mitochondrial membrane potentials. JC-1 selectively accumulates and aggregates in mitochondria, shifting its emission color from green to red as the membrane potential increases. A higher red-to-green fluorescence ratio (590:530) indicates a healthy or hyperpolarized mitochondrial membrane potential, whereas a lower ratio signifies depolarization.

**Figure 5:**
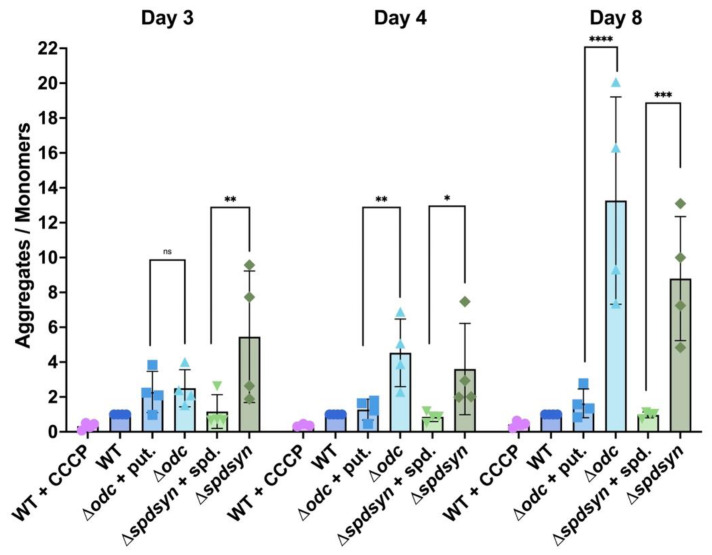
Mitochondrial membrane potential in wild-type and mutant parasites as monitored by JC-1 aggregate-to-monomer ratios. Wild-type parasites (WT), ∆*odc* mutants cultured in either 100 µM putrescine-supplemented or polyamine-free media, and ∆*spdsyn* mutants cultured in either 100 µM spermidine-supplemented or polyamine-free media were analyzed after 3, 4, and 8 days. The 590:530 fluorescence ratio was calculated for each data point as the aggregate-to-monomer ratio. The wild-type aggregate-to-monomer ratio was set to 1 as a baseline, allowing for the ratios of other cell lines and conditions to be normalized relative to this reference. The experiment was performed four times (*n* = 4). Statistical significance is represented as follows: ns (not significant), * *p* ≤ 0.05, ** *p* ≤ 0.01, *** *p* ≤ 0.001, and **** *p* ≤ 0.0001.

We validated our method using CCCP, a known mitochondrial uncoupler. As expected, CCCP treatment caused a shift from red to green fluorescence, resulting in ~60% lower aggregate-to-monomer ratio in wild-type cells (Figure 5). Throughout the experiment, the aggregate-to-monomer ratios of the ∆*odc* and ∆*spdsyn* mutants in media supplemented with putrescine or spermidine, respectively, remained similar to that of wild-type parasites, with minor but insignificant variability observed (Figure 5).

In contrast, the ∆*odc* parasites incubated in polyamine-free media showed an increased aggregate-to-monomer ratio compared to those supplemented with putrescine (Figure 5). Although the ratios were similar between the two groups on day 3, a significant difference appeared on day 4 (*p* = 0.0038) and became even more pronounced by day 8 (*p* ≤ 0.0001). A comparable increase in aggregate-to-monomer ratio was observed in ∆*spdsyn* mutants in polyamine-free media relative to those in spermidine-supplemented media across all sample days (*p* = 0.0020 on day 3, *p* = 0.0138 on day 4, and *p* = 0.0008 on day 8). The observed elevated aggregate-to-monomer ratio in polyamine-starved ∆*odc* and ∆*spdsyn* mutants indicates hyperpolarized mitochondrial membranes.

Overall, these results suggest that both mutant cell lines exhibited altered mitochondrial membrane potential in polyamine-free conditions compared to wild-type and supplemented mutants.

### 3.5. Putrescine Depletion Triggers DNA Fragmentation

Because mitochondrial dysfunction can be a sign of apoptosis, we investigated if DNA fragmentation, a hallmark of apoptosis, occurred in the polyamine-starved cell lines. A TUNEL assay was used to detect free hydroxyl ends produced during DNA degradation by endonucleases.

**Figure 6:**
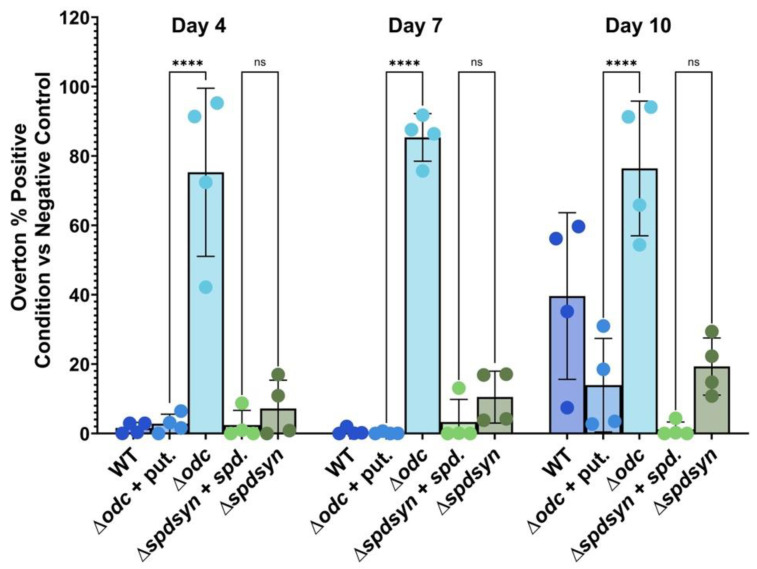
Percentage of cells with DNA fragmentation assessed by flow cytometry and TUNEL analysis. Wild-type parasites (WT), ∆*odc* mutants cultured in either 100 µM putrescine-supplemented or polyamine-free media, and ∆*spdsyn* mutants cultured in either 100 µM spermidine-supplemented or polyamine-free media were analyzed. Each cell line was analyzed after 4, 7, and 10 days. The experiment was performed four times (*n* = 4). Statistical significance is represented as follows: ns (not significant) and **** *p* ≤ 0.0001.

Wild-type cells showed no DNA degradation on day 4 or 7, but exhibited DNA degradation on day 10 when they experienced general nutrient deprivation (Figure 6). Some degradation was also observed by day 10 in the supplemented ∆*odc* mutants but surprisingly minimal DNA degradation was seen in the supplemented ∆*spdsyn* parasites. Notably, on day 10, the percentage of DNA degradation in wild-type parasites was significantly higher compared to that of supplemented ∆*spdsyn* mutants (*p* = 0.003).

DNA fragmentation was markedly increased in ∆*odc* mutants incubated in polyamine-free media throughout the entire incubation period (Figure 6). In comparison to ∆*odc* mutants supplemented with polyamines, the difference was highly significant, with *p* < 0.0001 observed on days 4, 7, and 10. In contrast, ∆*spdsyn* mutants exhibited only a slight statistically insignificant increase in DNA degradation compared to their counterparts incubated in spermidine-supplemented media.

To summarize, ∆*odc* mutants incubated in polyamine-free media exhibited consistently high levels of DNA fragmentation throughout the experiment, strikingly exceeding those observed in their supplemented counterparts. Remarkably, the ∆*spdsyn* mutants supplemented with spermidine demonstrated minimal DNA fragmentation, even at day 10—a time point by which substantial DNA degradation was evident across all other cell lines and conditions.

### 3.6. Polyamine Deprivation Causes Membrane Modifications

**Figure 7:**
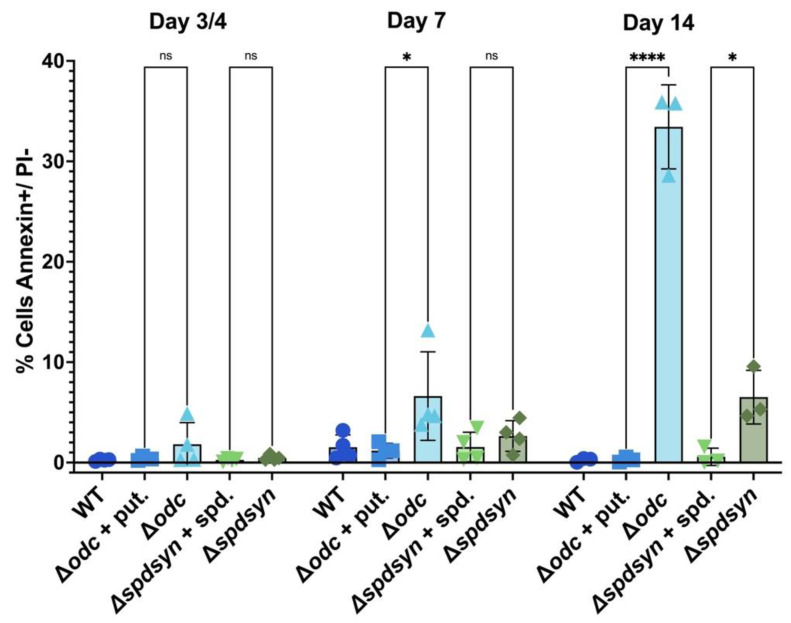
Percent of apoptotic cells as assessed by Annexin V and propidium iodide (PI) staining. The percentage of parasites that stained positive for Annexin V (indicating early apoptosis) and negative for PI (indicating membrane integrity) is shown. Wild-type parasites (WT), ∆*odc* mutants in either 100 µM putrescine-supplemented or polyamine-free media, and ∆*spdsyn* mutants in either 100 µM spermidine-supplemented or polyamine-free media were analyzed. Samples were collected on days 3 or 4, 7, and 14 of the experiment. The experiment was performed four times (*n* = 4). Statistical significance is represented as follows: ns (not significant), * *p* ≤ 0.05, and **** *p* ≤ 0.0001.

We used Annexin V and PI staining in flow cytometry to differentiate live, apoptotic, necrotic, and late apoptotic/necrotic cells based on membrane integrity and phosphatidylserine (PS) exposure. Wild-type cells, ∆*odc* mutants (with or without putrescine supplementation), and ∆*spdsyn* mutants (with or without spermidine supplementation) were incubated for 14 days, with samples analyzed on day 3 or 4, day 7, and day 14. On day 3 or 4, less than 2% of cells were Annexin V-positive and PI-negative, indicating minimal apoptosis (Figure 7, Table 1). By day 7, the percentage of apoptotic cells remained low (below 3%) in wild-type cells, putrescine-supplemented ∆*odc* mutants, and ∆*spdsyn* mutants, regardless of spermidine supplementation. In contrast, ∆*odc* mutants grown in polyamine-free media showed a significantly (*p* = 0.0064) higher rate of apoptosis at 6.62%, compared to 1.19% in putrescine-supplemented ∆*odc* mutants. This difference was even more profound by day 14 (*p* < 0.0001), with ∆*odc* mutants grown in polyamine-free media exhibiting 33.43% apoptotic cells, compared to a low percentage of 0.29% in supplemented ∆*odc* mutants. A more modest but statistically significant (*p* = 0.0278) increase in apoptosis was observed in ∆*spdsyn* mutants incubated in polyamine-free media, 6.52%, compared to those with spermidine supplementation, where apoptosis remained at 0.58%.

Analysis of Annexin V-negative PI-negative cells, indicating live parasites, revealed a notably high percentage of live cells, 72.73%, in ∆*spdsyn* mutants incubated in polyamine-free media at day 14 (Table 1). In contrast, wild-type cells and ∆*spdsyn* and ∆*odc* mutants grown with polyamine supplementation showed less than 10% in live cells, with ∆*odc* mutants incubated in polyamine-free media displaying 15.68% in PI-negative cells.

Collectively, flow cytometry analysis using Annexin V and PI staining showed that ∆*odc* mutants incubated in polyamine-free media had significantly higher levels of apoptosis over time compared to mutants with polyamine supplementation. In addition, ∆*spdsyn* mutants in polyamine-free media displayed a much higher percentage of live cells at day 14 than wild-type cells and supplemented mutants.

## 4. Discussion

Although recent studies have underscored the critical nature of polyamines in *Leishmania* parasites [28,36,44,74,75,76], their specific functions remain largely unexplored. Putrescine has emerged as a critical metabolite that has essential functions beyond its role as precursor for spermidine formation [71,77]. *L. donovani* polyamine pathway mutants provide valuable tools to investigate the roles of putrescine, since, under polyamine-free conditions, residual spermidine levels remain comparably low in both lines, while putrescine depletes rapidly in ∆*odc* mutants and accumulates in ∆*spdsyn* mutants [71].

Our findings suggest that putrescine depletion has a direct impact on growth, replication, metabolism, and type of cell death. The depletion of putrescine in the ∆*odc* mutants incubated in polyamine-free media led to an immediate cessation of proliferation and DNA replication upon polyamine withdrawal, while ∆*spdsyn* mutants grown in polyamine-free media presented a less severe growth and replication impairment (Figure 2 and Figure 3). Notably, ∆*odc* mutants exhibited a substantial metabolic decline, unlike the ∆*spdsyn* mutants (Figure 4), suggesting a more critical role for putrescine in cellular metabolism. Although both mutants showed a hyperpolarization of the mitochondrial membrane potential in polyamine-free media (Figure 5), only the ∆*odc* cells exhibited a significant increase in the apoptosis-like indicators, DNA fragmentation and Annexin V staining (Figure 6 and Figure 7).

**Figure 8:**
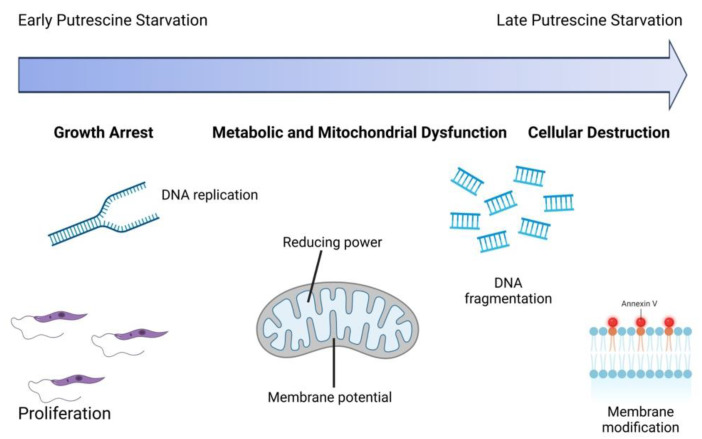
Model of cellular impairments due to putrescine depletion over time. Putrescine depletion in ∆*odc* mutants led to profound cellular health impairments. DNA replication and cell proliferation ceased immediately upon polyamine withdrawal. By day 3, mitochondrial dysfunction was evident, marked by a decrease in reductive capacity and mitochondrial membrane hyperpolarization. DNA fragmentation was observed on day 4, and membrane modifications began on day 7 and progressively worsened, ultimately leading to an apoptosis-like cell death. Created in BioRender. Johnston, J. (2024) https://BioRender.com/q18x404 (accessed on 17 December 2024).

Cell growth data over 11 days (Figure 2A) insinuate that the elevated polyamine levels present in the supplemented ∆*odc* and ∆*spdsyn* mutants (Figure 8) [71] may provide protection and bolster cell survival during stress conditions. Our results show that in the second week of incubation, the supplemented ∆*odc* and ∆*spdsyn* mutants had slightly higher cell numbers compared to wild-type parasites, suggesting greater resilience under nutrient-depleted conditions. This trend was further supported by the absence of PI staining (Table 1), which indicated that, by day 14, only 0.19% of wild-type cells remained viable, whereas 4.39% and 9.93% of the supplemented ∆*odc* and ∆*spdsyn* mutants, respectively, were still alive. However, adding polyamines (either 500 µM putrescine, 500 µM spermidine, or both) to the media did not increase the cell numbers of wild-type parasites (Figure 2C). This might indicate that wild-type parasites, which can synthesize adequate polyamines for their growth and survival, do not readily import additional polyamines. While *Leishmania* parasites possess polyamine transporters [78,79,80,81], and the polyamine pathway mutants rely on these transport systems for survival (Figure 2) [43,44,45,46,71], little is known about the regulation of polyamine transport. An alternative explanation for the enhanced resilience in the supplemented mutants could be other cellular or metabolic adaptations that occurred due to gene deletion events.

Previous studies have shown that ∆*spdsyn* parasites can enter a quiescent-like state and survive for up to six weeks in polyamine-free media [71]. Our findings support these observations, as we found that a substantial proportion of ∆*spdsyn* parasites remained viable even after prolonged incubation in polyamine-free media, as indicated by lack of PI staining (Table 1). Specifically, after 14 days, 72.73% of the cells were alive, a much higher percentage than observed in wild-type parasites, spermidine-supplemented ∆*spdsyn* mutants, or in ∆*odc* parasites, regardless of putrescine supplementation.

The persistence or quiescence of *Leishmania* and related parasites has gained attention due to its links to treatment failure, relapse, and chronic disease [20,21,22,23,82,83,84]. Understanding quiescence mechanisms could lead to better treatment paradigms and reduced relapse rates. Both supplemented ∆*odc* and ∆*spdsyn* mutants and ∆*spdsyn* mutants incubated in polyamine-free media exhibited persistence-like traits, characterized by stable cell numbers (Figure 2) and/or higher percentages of viable cells (Table 1). These traits were associated with significantly elevated putrescine levels, but variable spermidine pools [71]. Specifically, supplemented ∆*odc* mutants show roughly twice the putrescine levels of wild-type cells, but similar spermidine levels. In contrast, supplemented ∆*spdsyn* mutants have about three times as much putrescine and twice as much spermidine as wild-type parasites. Meanwhile, ∆*spdsyn* parasites incubated in polyamine-free media display reduced spermidine levels, but their putrescine content is five times higher than that of wild-type parasites. Taken together, these findings suggest that elevated putrescine levels may play a role in promoting parasite persistence. In contrast, the ∆*odc* mutants in polyamine-free media, which do not persist in culture, exhibit undetectable levels of putrescine.

The ability of mutant parasites to proliferate in polyamine-free media (Figure 2) closely aligned with their replication profile (Figure 3). The ∆*odc* mutants were unable to synthesize DNA and showed no cell growth, while the ∆*spdsyn* mutants exhibited limited DNA replication and low levels of proliferation. These findings suggest that putrescine is important for DNA synthesis. Because DNA replication is required for cell division, this impairment alone could account for the growth deficit observed in the putrescine-depleted ∆*odc* mutants (Figure 2).

To evaluate metabolic activity in the mutant cell lines, we used resazurin assays, which measure the reduction in resazurin to the fluorescent compound resorufin by cellular dehydrogenases in the presence of NADH or NADPH [85,86]. This reaction serves as an indirect indicator of mitochondrial health, as the majority of NADH is typically produced in mitochondria through energy-generating pathways such as the TCA cycle and oxidative phosphorylation [87,88]. The ∆*odc* parasites cultured in polyamine-free media exhibited significantly reduced metabolic activity compared to wild-type parasites and supplemented ∆*odc* mutants (Figure 4). In contrast, ∆*spdsyn* parasites showed metabolic activity comparable to wild-type parasites, regardless of polyamine supplementation (Figure 4). These findings suggest that reduced spermidine levels in ∆*spdsyn* mutants do not compromise cellular and mitochondrial metabolisms. However, maintaining intracellular levels of putrescine appears to be important for metabolic activity, overall cell health, and potentially mitochondrial function.

A critical role of putrescine in mitochondrial function and integrity in *Leishmania* parasites has previously been reported. The ODC inhibitor 1, 4-diamino-2-butanone reduces intracellular polyamine levels and causes structural and functional mitochondrial damage in both *Leishmania* and the related parasite *Trypanosoma cruzi* [58,89]. Notably, trypanosomatid parasites, like *Leishmania*, are especially vulnerable to mitochondrial dysfunction because they have only a single mitochondrion per cell, making this organelle critical for parasite survival and a potential target for therapeutic intervention [90,91].

To further investigate whether putrescine depletion in ∆*odc* mutants impacts mitochondrial function, we assessed mitochondrial membrane potential using JC-1, a cationic membrane-permeable dye commonly employed for this purpose [92,93,94,95]. Both mutant cell lines exhibited an altered mitochondrial membrane potential under polyamine-free conditions compared to wild-type and supplemented mutants, with a higher aggregate-to-monomer ratio indicative of hyperpolarization (Figure 5). While mitochondrial depolarization is often linked to dysfunction, as it disrupts ion gradients and ATP production, hyperpolarization can also signal stress and contribute to cell death. A recent publication reported that hypericin, whose main mechanism of action is the inhibition of SPDSYN, induces mitochondrial membrane hyperpolarization and cell death in *L. donovani* [76]. Mitochondrial hyperpolarization, induced by the inhibition of F0-F1 ATP synthase or complex I, has been shown to cause increased reactive oxygen species production and programmed cell death in *Leishmania* parasites [96,97,98].

Reduced metabolic activity was observed exclusively in putrescine-depleted ∆*odc* mutants, but not in ∆*spdsyn* mutants, when both were incubated in polyamine-free media (Figure 4). In contrast, mitochondrial hyperpolarization was observed in both mutant cell lines under these conditions (Figure 5). This observation suggests that mitochondrial hyperpolarization is likely driven by the low spermidine levels shared by both mutants. Spermidine may play an important role in sustaining mitochondrial membrane potential through electron transport chain activity, while putrescine appears to be critical for maintaining cellular reducing power, potentially through its influence on NAD(P)H levels and/or NAD(P)H dehydrogenase activity.

In mammalian cells, spermidine has been shown to directly enhance mitochondrial health by improving mitochondrial respiration, membrane potential, and ATP production [99,100,101,102]. Additionally, spermidine-mediated hypusination of eIF5A plays a crucial role in maintaining mitochondrial function, as lower levels of hypusinated eIF5A are associated with reduced oxygen consumption and ATP generation [103,104,105]. While it is unknown to what extent these mechanisms occur in *Leishmania* parasites, the low spermidine levels in the mutant cell lines likely lead to reduced eIF5A hypusination, which may in turn contribute to the impairment of mitochondrial health and respiration in a similar manner to mammalian cells.

Because mitochondrial dysfunction can lead to an apoptosis-like phenotype [98,106,107,108], we examined other hallmarks of apoptosis. DNA fragmentation and membrane modifications, driven by endonuclease-mediated cleavage of chromosomal DNA and the externalization of phospholipids such as phosphatidylserine, are central features of apoptosis, and both of these mechanisms have been previously described in *Leishmania* parasites [109,110,111,112]. While phosphatidylserine itself has been reported to be absent in *Leishmania* parasites, similar phospholipids appear to perform an analogous role, as Annexin V staining—used to detect phosphatidylserine externalization—has been observed in numerous studies of these organisms [110,111,112,113].

DNA fragmentation was detected within the first week of incubating ∆*odc* mutants in polyamine-free media (Figure 6), followed by membrane modifications observed during the second week of putrescine starvation (Figure 7). These findings suggest that putrescine depletion induced an apoptosis-like death phenotype. While both ∆*odc* and ∆*spdsyn* mutants incubated in polyamine-free media underwent mitochondrial hyperpolarization (Figure 5), the apoptosis phenotype was only observed in the ∆*odc* mutants, perhaps indicating that the elevated putrescine levels in the ∆*spdsyn* mutants protected the cells from undergoing programmed cell death. Similarly, supplemented ∆*spdsyn* mutants demonstrated significantly lower levels of DNA fragmentation after 10 days of incubation compared to other cell lines and conditions (Figure 6). This protective effect may have been due to the elevated intracellular polyamine levels.

The concept of programmed cell death in single-cell protozoan parasites like *Leishmania* remains intriguing and controversial, given that a single-celled organism may not seem to require such a process [109,110,112,114]. Current hypotheses include the idea that apoptosis may benefit the population as a whole by avoiding hyperparasitism or the immune response in the host [109,110,114,115,116]. Although *Leishmania* lacks classic apoptotic proteins, and signaling pathways have not been identified, the parasite shows evidence of apoptosis-like characteristics, including mitochondrial dysfunction, DNA fragmentation, membrane modifications, cell shrinkage, and rounding [109,110,111,112,114]. This insinuates that apoptosis in *Leishmania* and other protozoan parasites may represent a rudimentary or primitive evolutionary precursor to the more complex forms of cell death observed in multicellular organisms [109,116,117]. Nonetheless, further research into *Leishmania* apoptosis holds promise for uncovering novel targets for drug development and therapeutic intervention [109,110]. The ∆*odc* cell line, in particular, may serve as a valuable tool for exploring the molecular mechanisms underlying programmed cell death. Notably, most prior studies have focused on apoptosis induced by drugs, whereas gene deletion mutants like ∆*odc*, which exhibit intrinsic apoptotic features, could provide a more precise and controlled model for understanding these processes.

The limitations of the studies presented here include uncertainty about whether the observed effects are directly linked to putrescine depletion or occur as a result of cellular stress induced by putrescine starvation. Additionally, there was some inherent variability across cell lines and conditions, particularly in the DNA fragmentation and membrane modification assays. This variability may result from polyamine-starved parasites forming a heterogeneous population, with individual cells exhibiting distinct responses. Furthermore, these studies were conducted in the promastigote stage, highlighting the need for future research in the medically relevant amastigote stage.

## 5. Conclusions

Our findings, combined with previously published observations [71], provide a better understanding of the functions of putrescine. A key insight is that putrescine depletion triggers both early and late cellular changes (Figure 8). Early changes, observed within the first two days of starvation, include growth arrest, cessation of DNA replication, and morphological alterations, followed by reduced metabolism, mitochondrial dysfunction, and DNA fragmentation. By the second week of starvation, membrane modifications, another hallmark of an apoptosis-like cell death, emerged. Notably, these effects are specific to putrescine depletion and are not observed with general nutrient starvation. Together, these findings highlight the essential roles of putrescine in DNA replication, cellular proliferation, and metabolism. Moreover, they offer a plausible explanation for the more pronounced effects of the ∆*odc* gene deletion on in vivo infectivity compared to deletions in other polyamine pathway enzymes [44,45,77]. In conclusion, our studies support the idea that the polyamine biosynthetic pathway in *Leishmania* is a promising therapeutic target, with ODC standing out as a key target for therapeutic development.

## Figures and Tables

**Table 1:** Percentage of parent cell population in each gated quadrant of Annexin V/propidium iodide (PI) double staining.

Day	Cell Line	Apoptotic (AnnexinV+/PI−)	Live (AnnexinV−/PI−)	Late Apoptosis/Necrotic (AnnexinV+/PI+)	Necrotic (AnnexinV−/PI+)
**Day 3–4**	**WT**	0.25 ± 0.09	**96.78 ± 1.85**	1.56 ± 0.62	1.43 ± 1.31
	**Δ*odc* + put.**	0.4 ± 0.16	**97.48 ± 0.49**	1.26 ± 0.64	0.9 ± 0.28
	**Δ*odc***	1.82 ± 1.87	**88.28 ± 9.81**	6.73 ± 7.61	3.19 ± 2.39
	**Δ*spdsyn* + spd.**	0.26 ± 0.14	**97.8 ± 0.64**	1 ± 0.21	0.96 ± 0.59
	**Δ*spdsyn***	0.47 ± 0.23	**96.13 ± 0.34**	2.03 ± 0.73	1.38 ± 0.91
**Day 7**	**WT**	1.52 ± 1.08	**77.1 ± 12.09**	16.21 ± 10.54	5.19 ± 1.7
	**Δ*odc* + put.**	1.19 ± 0.63	**68.73 ± 16.09**	19.34 ± 9.03	10.79 ± 7.84
	**Δ*odc***	6.62 ± 3.82	**74.6 ± 6.03**	13.13 ± 3.05	5.69 ± 1.84
	**Δ*spdsyn* + spd.**	1.55 ± 1.28	**68.13 ± 15.75**	24.7 ± 12.79	5.67 ± 3.94
	**Δ*spdsyn***	2.64 ± 1.32	**82.23 ± 8.03**	9.88 ± 7.82	5.27 ± 2.39
**Day 14**	**WT**	0.26 ± 0.17	0.19 ± 0.23	**87.23 ± 7.64**	12.33 ± 7.57
	**Δ*odc* + put.**	0.29 ± 0.2	4.39 ± 6.09	**78.53 ± 13.96**	16.75 ± 9.29
	**Δ*odc***	**33.43 ± 3.42**	15.68 ± 5.97	**37.1 ± 11.8**	13.75 ± 6.78
	**Δ*spdsyn* + spd.**	0.58 ± 0.7	9.93 ± 13.91	**79.3 ± 12.42**	10.19 ± 2.51
	**Δ*spdsyn***	6.52 ± 2.18	**72.73 ± 20.88**	13.34 ± 13.06	7.45 ± 5.83

Values are presented with standard deviation (*n* = 4). Bolded are the highest percentages within each cell line and condition on every sample day.

## Data Availability

The data presented in this study are available on request from the corresponding author due to the large volume or specialized format of the raw data, requiring specialized data sharing.

## Supplementary Materials

The following supporting information can be downloaded at: https://www.mdpi.com/article/10.3390/pathogens14020137/s1, Figure S1: Proliferation of wild-type and mutant parasites in response to polyamine availability.
