# Supplementary material for: Putrescine Depletion in Leishmania donovani Parasites Causes Immediate Proliferation Arrest Followed by an Apoptosis-like Cell Death
Source: Pathogens. 2025 Feb 2;14(2):137. doi: 10.3390/pathogens14020137 (PMC11858418; doi:10.3390/pathogens14020137)
Supplement: Supplementary file 1 [file pathogens-14-00137-s001.zip › pathogens-3410626-supplementary.pdf]

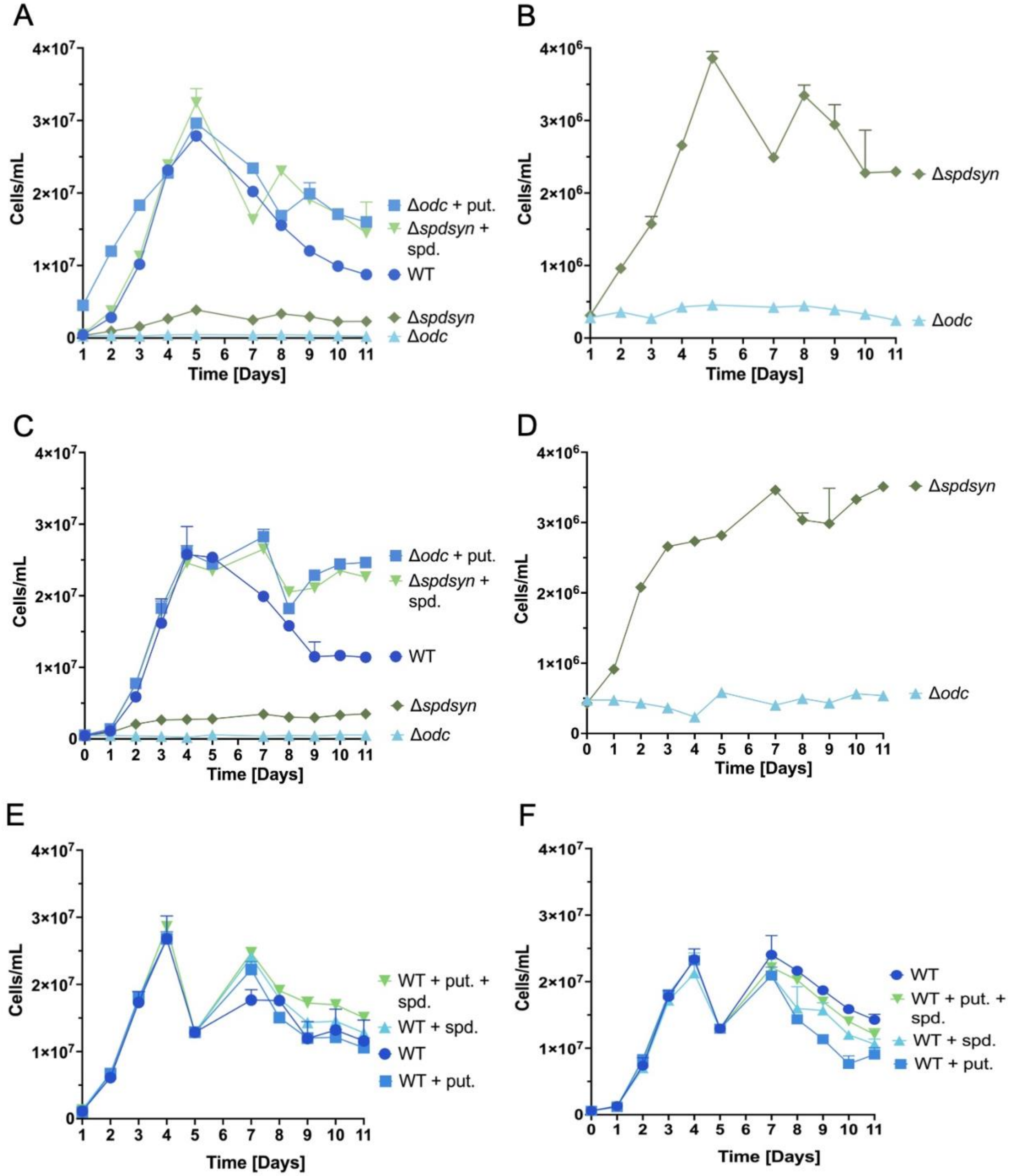

Supplemental Figure S1: Proliferation of wild-type and mutant parasites in response to polyamine availability. Parasites proliferation was monitored over 11 days using a flow cytometer. **(A and C)** Proliferation of wild-type (WT) cells in polyamine-free media (dark blue circles), Δ*odc* mutants in media with 100 μM putrescine (blue squares), Δ*spdsyn* mutants in media with 100 μM spermidine (green triangles), as well as Δ*odc* (light blue triangles) and Δ*spdsyn* mutants (dark green diamonds) in polyamine-free media. **(B and D)** Growth of the Δ*odc* (light blue triangles) and Δ*spdsyn* (dark green diamonds) mutants

grown in polyamine-free media is shown to allow a better comparison of the cellular proliferation rate between the two mutants. **(E** and **F)** Growth of wild-type parasites in polyamine-free media (dark blue circles), in media supplemented with 500 μM putrescine (blue squares), 500 μM spermidine (light blue triangles), or a combination of 500 μM putrescine and 500 μM spermidine (light green triangles). Three experiments were conducted in technical triplicate (n = 3) for each experimental design. Consistent results were observed across all experiments and two representative experiments from each design are displayed, in addition to the experiments shown in Figure 2.
